# A healthier day, a healthier mind: linking 24-h movement behavior guideline adherence to mental health in university students

**DOI:** 10.3389/fpsyg.2026.1889704

**Published:** 2026-09-23

**Authors:** Siwen Shan, Biao Zheng

**Affiliations:** 1 College of Physical Education, Hunan First Normal University, Changsha, Hunan, China; 2 College of Physical Education, Hunan Normal University, Changsha, Hunan, China

**Keywords:** 24-h movement behaviors, guideline adherence, mental health, university adaptation, university students

## Abstract

**Background:**

University students face substantial challenges to psychological well-being, and healthy daily movement behaviors may be relevant to positive mental health. This study examined the association between adherence to 24-h movement behavior guidelines and positive mental health among university students, as well as the hypothesized statistical indirect associations through university adaptation and psychological resilience.

**Methods:**

A cross-sectional survey was conducted among 1,856 undergraduate students from two universities in Hunan Province, China. Adherence to 24-h movement behavior guidelines was assessed based on physical activity, recreational screen behavior, and sleep duration. University adaptation, psychological resilience, and positive mental health were measured using self-report scales, with positive mental health operationalized using the Mental Health Continuum-Short Form (MHC-SF). Pearson correlation analysis and PROCESS Model 6 were used to estimate the hypothesized serial direct and indirect associations.

**Results:**

Complete adherence to all three guidelines was relatively low. Sleep duration had the highest adherence rate, whereas recreational screen behavior had the lowest. Adherence to more guidelines was positively associated with university adaptation, psychological resilience, and positive mental health; however, the correlations between guideline adherence and these variables were modest (*r* = 0.286–0.318). PROCESS Model 6 identified significant statistical indirect associations between guideline adherence and positive mental health through university adaptation and psychological resilience, both independently and sequentially.

**Conclusion:**

Greater breadth of 24-h movement behavior guideline adherence was modestly associated with better MHC-SF positive mental health among university students. University adaptation and psychological resilience showed statistical indirect associations consistent with the hypothesized pathways, but the cross-sectional design does not establish temporal mediation or causality.

## 1 Introduction

University life is an important transitional period during which students experience major changes in academic demands, interpersonal relationships, living arrangements, autonomy, and future career planning. Although this period provides opportunities for personal growth, university students are also vulnerable to psychological distress. Evidence from the WHO World Mental Health International College Student Project showed that mental health problems are common among college students, which may affect academic engagement, social relationships, and long-term development (Auerbach et al., 2018). Therefore, identifying modifiable lifestyle and psychosocial factors that contribute to university students’ mental health is an important issue in psychology, public health, and higher education.

Physical activity has been widely regarded as a protective factor for mental health. Recent evidence suggests that physical exercise can reduce symptoms of depression, anxiety, and stress and improve psychological well-being among university students (Liu et al., 2025). However, university students’ daily behaviors do not occur independently. Time spent in physical activity, sedentary behavior, screen use, and sleep is distributed within the same 24-h day. Thus, examining physical activity alone may not fully capture the lifestyle patterns that influence mental health.

The concept of 24-h movement behaviors provides an integrated framework for understanding health-related time-use behaviors. This framework emphasizes that physical activity, sedentary behavior, and sleep are interdependent components of a full 24-h period (Chaput et al., 2014; Ross et al., 2020). For adults aged 18–64 years, 24-h movement guidelines recommend a healthy combination of moderate-to-vigorous physical activity, light physical activity, limited sedentary behavior, and sufficient sleep (Ross et al., 2020). Prospective evidence also suggests broader health relevance of meeting a greater number of 24-h movement guidelines in adults (López-Gil et al., 2025). In the present manuscript, “24-hour movement behaviors” refers to the behaviors themselves, “24-hour movement guidelines” refers to recommended behavioral thresholds, and “guideline adherence” refers to whether and how many of the operationalized thresholds are met. Because the present study measured recreational screen behavior rather than total sedentary time, recreational screen exposure should not be interpreted as a complete measure of the sedentary-behavior component.

Previous studies have shown that 24-h movement behaviors and guideline adherence are associated with mental-health-related outcomes among university students. For example, Liang et al. (2021) found that meeting sleep recommendations was associated with lower depression and anxiety among Chinese college students. Wang et al. (2023) reported prospective associations between 24-h movement behavior compositions and depression, anxiety, and stress among overweight or obese college students. Zhang et al. (2024) further found that meeting more 24-h movement guidelines was associated with a lower risk of depressive symptoms, while Dong et al. (2025) used compositional data analysis to link 24-h movement behaviors with perceived stress. More recently, Li et al. (2026) showed in Chinese college students that the relative associations of moderate-to-vigorous physical activity, sedentary time, and sleep differed across depression, anxiety, and stress outcomes. Thus, the association between 24-h movement behaviors and mental-health-related outcomes is not itself new. The principal contribution of the present study is instead to examine university adaptation and psychological resilience as potential psychosocial correlates that statistically link guideline adherence with positive mental health.

University adaptation and psychological resilience may represent two theoretically plausible psychosocial factors associated with this relationship. University adaptation, also referred to as college adjustment, reflects students’ adjustment to academic, social, personal-emotional, and institutional aspects of university life (Baker and Siryk, 1984; Credé and Niehorster, 2012). Psychological resilience refers to the capacity to maintain or regain adaptive functioning when facing stress, adversity, or challenge (Masten, 2001; Connor and Davidson, 2003). Healthy movement guideline adherence may be associated with differences in daily functioning that are relevant to adjustment to university life, and better adaptation may in turn co-occur with greater resilience and positive mental health. The present study therefore tests a theoretically specified pattern of statistical indirect associations rather than temporal or causal mediation.

## 2 Literature review and research hypothesis

### 2.1 24-h movement behaviors and mental health

The 24-h movement behavior framework suggests that physical activity, sedentary behavior, and sleep should be considered together because they jointly compose the full day (Chaput et al., 2014; Ross et al., 2020). For university students, this perspective is especially useful because their daily routines often include long study-related sitting, frequent screen use, insufficient physical activity, and irregular sleep. A healthier combination of these behaviors may support emotional regulation, stress recovery, cognitive functioning, and social engagement.

Empirical evidence supports associations between 24-h movement behaviors and mental-health-related outcomes in university students. Liang et al. (2021) found that meeting 24-h movement recommendations, especially the sleep recommendation, was associated with lower depression and anxiety. Wang et al. (2023) showed that different compositions of movement behaviors were prospectively associated with depression, anxiety, and stress. Zhang et al. (2024) reported that meeting more 24-h movement guidelines was associated with a lower risk of depressive symptoms. These findings support the relevance of an integrated daily-movement perspective, while also indicating that the individual behaviors and their combinations may not be equivalent in their associations with health outcomes.

Recent work has further argued for moving beyond uniform threshold-based recommendations toward precision 24-h movement behavior recommendations, because health-optimal combinations may differ by population, outcome, and individual characteristics (Tremblay et al., 2024). Consistent with this perspective, the compositional analysis by Li et al. (2026) found outcome-specific associations of moderate-to-vigorous physical activity, sedentary time, and sleep with depression, anxiety, and stress among Chinese college students. Accordingly, the 0–3 adherence score used in the present study should be interpreted as the breadth of guideline attainment rather than the composition, quality, or optimality of the overall 24-h movement profile.

*H1*: Adherence to 24-h movement behavior guidelines is positively associated with positive mental health among university students.

### 2.2 University adaptation as a hypothesized indirect pathway

University adaptation refers to students’ adjustment to the academic, social, emotional, and institutional demands of university life. Students with better university adaptation are more likely to manage academic tasks, develop supportive peer relationships, experience a sense of belonging, and maintain emotional stability (Baker and Siryk, 1984; Credé and Niehorster, 2012). Poor university adaptation, in contrast, may increase loneliness, academic pressure, interpersonal difficulties, and psychological distress.

Adherence to 24-h movement behavior guidelines may be positively associated with university adaptation. Regular physical activity may be associated with greater opportunities for social interaction and campus participation; sufficient sleep may be associated with better attention, learning efficiency, and emotion regulation; and lower recreational screen exposure may be associated with greater engagement in academic and social activities. Importantly, recreational screen behavior is only one aspect of sedentary behavior and is not used here as a proxy for total sedentary time. Zhou et al. (2024) found that physical exercise was positively associated with college students’ social adaptation. In a different population, Gao et al. (2024) reported that meeting specific or integrated 24-h movement behavior guidelines was associated with academic engagement, resilience, and self-regulation in youth with internalizing problems. Although that evidence is not directly transferable to university students, it provides convergent support for a link between integrated movement behaviors and broader psychosocial functioning. Therefore, university adaptation was hypothesized to statistically account for part of the association between guideline adherence and positive mental health.

*H2*: A significant statistical indirect association is hypothesized between adherence to 24-h movement behavior guidelines and positive mental health through university adaptation.

### 2.3 Psychological resilience as a hypothesized indirect pathway

Psychological resilience is an important internal resource that helps individuals cope with stress, recover from setbacks, and maintain adaptive functioning (Masten, 2001; Connor and Davidson, 2003). University students often face academic pressure, interpersonal challenges, financial concerns, and career uncertainty. Students with higher resilience are more likely to regulate negative emotions, use effective coping strategies, and maintain better mental health.

Healthy movement behaviors may be associated with psychological resilience. Physical activity has been linked to self-efficacy, mastery experiences, lower stress, and positive affect, while sufficient sleep may be associated with emotional recovery and cognitive control. Lower recreational screen exposure may also coincide with more active engagement in daily life, although it should not be equated with lower total sedentary time. Gao et al. (2024) observed associations between meeting 24-h movement guidelines and resilience/self-regulation in youth with internalizing problems, while Guo (2025) reported a statistical mediation model involving physical exercise, mindfulness, psychological resilience, and university students’ mental health. These findings provide indirect support for resilience as a relevant psychosocial correlate, without establishing a temporal mechanism. Thus, psychological resilience was hypothesized to statistically account for part of the association between guideline adherence and positive mental health.

*H3*: A significant statistical indirect association is hypothesized between adherence to 24-h movement behavior guidelines and positive mental health through psychological resilience.

### 2.4 Hypothesized serial indirect pathway through university adaptation and psychological resilience

University adaptation and psychological resilience may also be statistically related in a sequential manner. University adaptation reflects students’ adjustment to the academic, interpersonal, emotional, and institutional demands of university life. Successful adaptation may be accompanied by repeated experiences of managing academic and social demands, stronger perceived competence, social support, and a sense of belonging; these contextual experiences are theoretically relevant to the development or expression of resilience when students encounter stress and adversity. Healthy movement guideline adherence may plausibly be associated with day-to-day functioning relevant to adaptation, whereas adaptation may in turn be associated with greater resilience. Evidence linking 24-h movement guideline adherence with academic engagement, resilience, and self-regulation provides indirect empirical support for this broader psychosocial linkage (Gao et al., 2024). On this basis, university adaptation was specified as the first intervening variable and psychological resilience as the second in the hypothesized serial model.

However, this ordering was specified *a priori* on theoretical grounds rather than established empirically. The reverse sequence is equally plausible: students with greater psychological resilience may cope more effectively with academic and interpersonal challenges and therefore adapt more successfully to university life. Reciprocal relationships are also possible. Because all variables were assessed concurrently, the present data cannot distinguish among these competing temporal sequences.

*H4*: A significant serial indirect association is hypothesized between adherence to 24-h movement behavior guidelines and positive mental health through university adaptation and psychological resilience. Specifically, greater guideline adherence is expected to be associated with better university adaptation, which is expected to be associated with higher psychological resilience and, in turn, better positive mental health.

## 3 Methods

### 3.1 Study design and participants

This study adopted a cross-sectional survey design to examine the association between adherence to 24-h movement behavior guidelines and positive mental health among university students, as well as the hypothesized statistical indirect associations through university adaptation and psychological resilience. Participants were recruited from Hunan First Normal University and Hunan Normal University in Hunan Province, China, using convenience sampling.

The inclusion criteria were as follows: (1) being a full-time undergraduate student at one of the two participating universities; (2) being aged 18 years or above; (3) being able to understand and complete the questionnaire independently; and (4) providing informed consent before participation. Participants were excluded if they submitted incomplete questionnaires, gave obviously patterned responses, completed the questionnaire in an unrealistically short time, or provided inconsistent demographic information.

A total of 2,381 questionnaires were distributed. After data screening according to the predefined exclusion criteria, 525 invalid questionnaires were excluded. Finally, 1,856 valid questionnaires were retained for analysis, yielding an effective response rate of 77.95%. The excluded questionnaires met one or more of the invalid-response criteria described above (Table 1).

**Table 1:** Questionnaire distribution and valid sample.

Characteristic	Category/statistic	Value
Sample flow		
Questionnaires distributed		2,381
Invalid questionnaires excluded		525
Valid questionnaires retained		1,856
Effective response rate		77.95%
Demographic characteristics		
Age, years		19.13 ± 0.56
Gender	Male	1,103
	Female	753
University	Hunan First Normal University	1,402
	Hunan Normal University	454
Academic year	First year	615
	Second year	831
	Third year	347
	Fourth year	63

The number of invalid questionnaires was calculated as the difference between distributed questionnaires and valid questionnaires.

### 3.2 Measures

#### 3.2.1 Demographic variables

Participants reported their gender, age, university, grade, major, place of residence, only-child status, family economic status, height, and weight. Body mass index (BMI) was calculated as weight in kilograms divided by height in meters squared. Gender, age, grade, university, major, residence, only-child status, family economic status, and BMI were included as covariates in the subsequent analyses.

#### 3.2.2 Adherence to 24-h movement behavior guidelines

Adherence to 24-h movement behavior guidelines was assessed based on three components: physical activity, recreational screen behavior, and sleep duration. The 24-h movement behavior framework emphasizes that physical activity, sedentary behavior, and sleep should be considered together within a complete 24-h day (Ross et al., 2020). The operational thresholds for the three components were based on the Canadian 24-Hour Movement Guidelines for Adults aged 18–64 years (Ross et al., 2020). As the present study focused on adult university students, this adult guideline framework was used as the reference standard for defining adherence.

Physical activity was assessed using the International Physical Activity Questionnaire-Short Form (IPAQ-SF; Craig et al., 2003). Participants reported the frequency and duration of moderate and vigorous physical activity during the past 7 days. The physical activity criterion was coded as met if the participant accumulated at least 150 min of moderate-to-vigorous physical activity per week, consistent with the adult 24-h movement guidelines (Ross et al., 2020).

Recreational screen behavior was assessed using items adapted from the Sedentary Behavior Questionnaire (SBQ; Rosenberg et al., 2010). Participants reported their average daily recreational screen time on weekdays and weekends, excluding time spent on academic tasks. Weighted average daily recreational screen time was calculated as [(weekday screen time x 5) + (weekend screen time x 2)]/7. The recreational screen-time criterion was coded as met if recreational screen time was no more than 3 h per day, corresponding to the recreational screen-time recommendation within the sedentary-behavior component of the adult 24-h movement guidelines (Ross et al., 2020). Because the present study assessed recreational screen time rather than total sedentary time, this threshold operationalized the recreational screen-time component of the broader sedentary-behavior recommendation.

Sleep duration was assessed using the sleep-duration item from the Pittsburgh Sleep Quality Index (PSQI; Buysse et al., 1989). Participants reported their usual nightly sleep duration during the past month. The sleep criterion was coded as met if the participant reported sleeping 7 to 9 h per night, consistent with the sleep-duration recommendation for adults aged 18–64 years in the 24-h movement guidelines (Ross et al., 2020).

Each component was coded as 0 = not meeting the guideline and 1 = meeting the guideline. The three components were summed to obtain the total number of 24-h movement behavior guidelines met, ranging from 0 to 3. A higher score indicated that the participant met more operationalized guideline thresholds. This score was used as the independent variable in the serial indirect-effects model. The additive score provides a parsimonious indicator of the breadth of guideline adherence and assigns equal weight to physical activity, recreational screen behavior, and sleep. This operationalization therefore assumes equal contributions and additive accumulation across the three components. It should not be interpreted as a compositional measure, an index of the quality of the full 24-h profile, or evidence that different behavioral configurations with the same score are equivalent. These limitations are particularly relevant in light of recent precision and compositional approaches to 24-h movement behaviors (Tremblay et al., 2024; Li et al., 2026) (Table 2).

**Table 2:** Coding criteria for 24-h movement behavior guideline adherence.

Component	Measurement tool	Criterion for meeting the guideline	Coding
Physical activity	IPAQ-SF	>=150 min of moderate-to-vigorous physical activity per week	0 = not met; 1 = met
Recreational screen behavior	Adapted SBQ items	<=3 h of recreational screen time per day	0 = not met; 1 = met
Sleep duration	PSQI sleep-duration item	7–9 h of sleep per night	0 = not met; 1 = met
Total adherence score	Sum of three components	Range: 0–3 guidelines met	Higher scores indicate more guidelines met

#### 3.2.3 University adaptation

University adaptation was assessed using a 24-item College Students’ Adaptability Scale used in recent Chinese college-student research (Ren et al., 2024). Compared with the original 67-item Student Adaptation to College Questionnaire (Baker and Siryk, 1989), this shorter measure is more suitable for a multi-scale survey because it reduces participant burden while still capturing key adaptation domains.

The scale includes six dimensions: learning adaptability, professional adaptability, homesickness adaptability, interpersonal adaptability, emotional adaptability, and economic adaptability, with four items for each dimension. Items were rated on a 5-point Likert scale ranging from 1 = strongly disagree to 5 = strongly agree. After reverse coding negatively worded items where applicable, item scores were averaged to create a total university adaptation score. Higher scores indicated better adaptation to university life. The internal consistency reliability of this scale was examined using Cronbach’s alpha in the final analytic sample.

#### 3.2.4 Psychological resilience

Psychological resilience was assessed using the Connor-Davidson Resilience Scale (CD-RISC; Connor and Davidson, 2003). The Chinese version of the scale has been widely used and has demonstrated acceptable psychometric properties among Chinese populations (Yu and Zhang, 2007). The scale contains 25 items measuring individuals’ ability to cope with stress, recover from adversity, maintain emotional stability, and adapt positively when facing challenges.

Each item was rated on a 5-point Likert scale ranging from 0 = not true at all to 4 = true nearly all the time. Higher total scores indicated higher psychological resilience. In this study, psychological resilience was specified as the second intervening variable in the serial indirect-effects model. The internal consistency reliability of this scale was examined using Cronbach’s alpha in the final analytic sample.

#### 3.2.5 Mental health

Mental health was measured using the Mental Health Continuum-Short Form (MHC-SF; Keyes et al., 2008). The MHC-SF assesses positive mental health from three dimensions: emotional well-being, social well-being, and psychological well-being. The Chinese version of the MHC-SF has shown good reliability and validity in Chinese samples (Guo et al., 2015). Accordingly, “mental health” in the present study refers specifically to positive mental health/well-being as operationalized by the MHC-SF and should not be interpreted as the absence of depression, anxiety, or other psychiatric symptoms.

The scale contains 14 items. Participants rated how often they experienced each positive mental health state during the past month on a 6-point frequency scale ranging from 0 = never to 5 = every day. Higher scores indicated better positive mental health. In this study, the total MHC-SF score was used as the dependent variable. The internal consistency reliability of this scale was examined using Cronbach’s alpha in the final analytic sample (Table 3).

**Table 3:** Measurement instruments.

Variable	Instrument	Main dimensions or content	Items	Scoring/use
Physical activity	IPAQ-SF	Moderate and vigorous physical activity	7	Used to calculate weekly MVPA
Recreational screen behavior	Adapted SBQ items	Weekday and weekend recreational screen time	2	Weighted daily average screen time
Sleep duration	PSQI sleep-duration item	Usual nightly sleep duration	1	Hours of sleep per night
University adaptation	24-item College Students’ Adaptability Scale	Learning, professional, homesickness, interpersonal, emotional, and economic adaptability	24	Higher scores indicate better adaptation
Psychological resilience	Connor-Davidson Resilience Scale	Coping with stress and recovering from adversity	25	Higher scores indicate greater resilience
Positive mental health	Mental Health Continuum-Short Form	Emotional, social, and psychological well-being	14	Higher scores indicate better mental health

Internal consistency reliability for the multi-item scales was evaluated using Cronbach’s alpha in the final analytic sample.

### 3.3 Data quality control and common method bias

Several procedures were used to ensure data quality. First, all questionnaires were completed anonymously to reduce social desirability bias. Second, participants were informed that there were no right or wrong answers and were encouraged to respond honestly. Third, invalid questionnaires were removed according to predefined criteria, including incomplete responses, extremely short completion time, patterned answers, and inconsistent demographic information. Fourth, the questionnaire sections were arranged to reduce participants’ awareness of the hypothesized relationships among variables.

Because all variables were collected using self-reported questionnaires, common method bias was considered. Procedural controls included anonymous responses, neutral instructions, and separation of predictor, intervening-variable, and outcome measures into different questionnaire sections. In the statistical analysis stage, Harman’s single-factor test was conducted to examine potential common method bias (Podsakoff et al., 2003).

### 3.4 Statistical analysis

All statistical analyses were conducted using SPSS 26.0 and the PROCESS macro. PROCESS is a regression-based tool commonly used for estimating direct and indirect effects in mediation and serial mediation models (Hayes, 2022).

First, descriptive statistics were used to summarize participants’ demographic characteristics and the distribution of 24-h movement behavior guideline adherence. Frequencies and percentages were reported for categorical variables, while means and standard deviations were reported for continuous variables. Second, Cronbach’s alpha coefficients were calculated to examine the internal consistency reliability of the university adaptation, psychological resilience, and MHC-SF positive mental health scales. A Cronbach’s alpha value above 0.70 was considered acceptable. Third, Pearson correlation analysis was conducted to examine the associations among guideline adherence, university adaptation, psychological resilience, and positive mental health.

Fourth, a serial indirect-effects model was estimated using PROCESS Model 6. In this model, the total number of 24-h movement behavior guidelines met was entered as the independent variable, university adaptation was specified as the first intervening variable (M1), psychological resilience as the second intervening variable (M2), and MHC-SF positive mental health as the dependent variable. The ordering of these variables was specified *a priori* on theoretical grounds. Gender, age, grade, university, major, residence, only-child status, family economic status, and BMI were included as covariates.

Although the adherence score was an ordered variable ranging from 0 to 3, it was treated as a continuous count variable in the main analysis because it represented the number of guideline thresholds met. This approach estimates whether broader guideline adherence is associated with better university adaptation, higher psychological resilience, and better positive mental health; it does not estimate the effects of specific behavioral compositions. As a robustness check, the adherence score was also dummy-coded into four categories, with meeting no guideline as the reference group. To facilitate comparison of the relative magnitude of the principal continuous regression paths, standardized regression coefficients (β) were additionally calculated as B × SDpredictor/SDoutcome using the sample standard deviations reported in Table 4.

**Table 4:** Descriptive statistics, reliability, and correlations among main variables.

Variable	*M*	SD	Cronbach’s α	1	2	3	4
1. 24-h movement behavior guideline adherence	1.33	0.93	–	1			
2. University adaptation	3.56	0.62	0.821	0.318**	1		
3. Psychological resilience	2.74	0.58	0.834	0.286**	0.542**	1	
4. Positive mental health	3.61	0.67	0.747	0.301**	0.496**	0.573**	1

M = mean; SD = standard deviation. **p < 0.01.

The indirect effects were tested using the bootstrap method with 5,000 resamples. Bias-corrected 95% confidence intervals were calculated. An indirect effect was considered statistically significant if the 95% confidence interval did not include zero. The significance level was set at *p* < 0.05. Given the cross-sectional design and concurrent assessment of the predictor, intervening variables, and outcome, PROCESS estimates were interpreted strictly as model-based direct and indirect statistical associations. They do not establish temporal precedence, causal mediation, or a mechanistic pathway.

## 4 Results

### 4.1 Common method bias test

Because all core variables were collected using self-reported questionnaires, Harman’s single-factor test was conducted to examine the potential influence of common method bias. The results showed that several factors had eigenvalues greater than 1. The first unrotated factor explained 26.84% of the total variance, which was below the commonly used threshold of 40%. This result suggested that no single factor accounted for the majority of the covariance among the measured variables. However, the Harman single-factor test is only a limited diagnostic and cannot establish the absence of common method variance. Because the principal constructs were assessed through self-report within the same survey, common method bias remains possible and should be considered when interpreting the observed associations.

### 4.2 Descriptive statistics and adherence rates

Figure 1 illustrates the questionnaire screening process and the final analytic sample. Of the 2,381 questionnaires distributed, 525 were excluded during data screening, including 152 because of incomplete responses, 98 because of patterned responses, 271 because of extremely short completion time, and 4 because of inconsistent demographic information. The remaining 1,856 questionnaires were retained for analysis, corresponding to an effective response rate of 77.95%.

**Figure 1:**
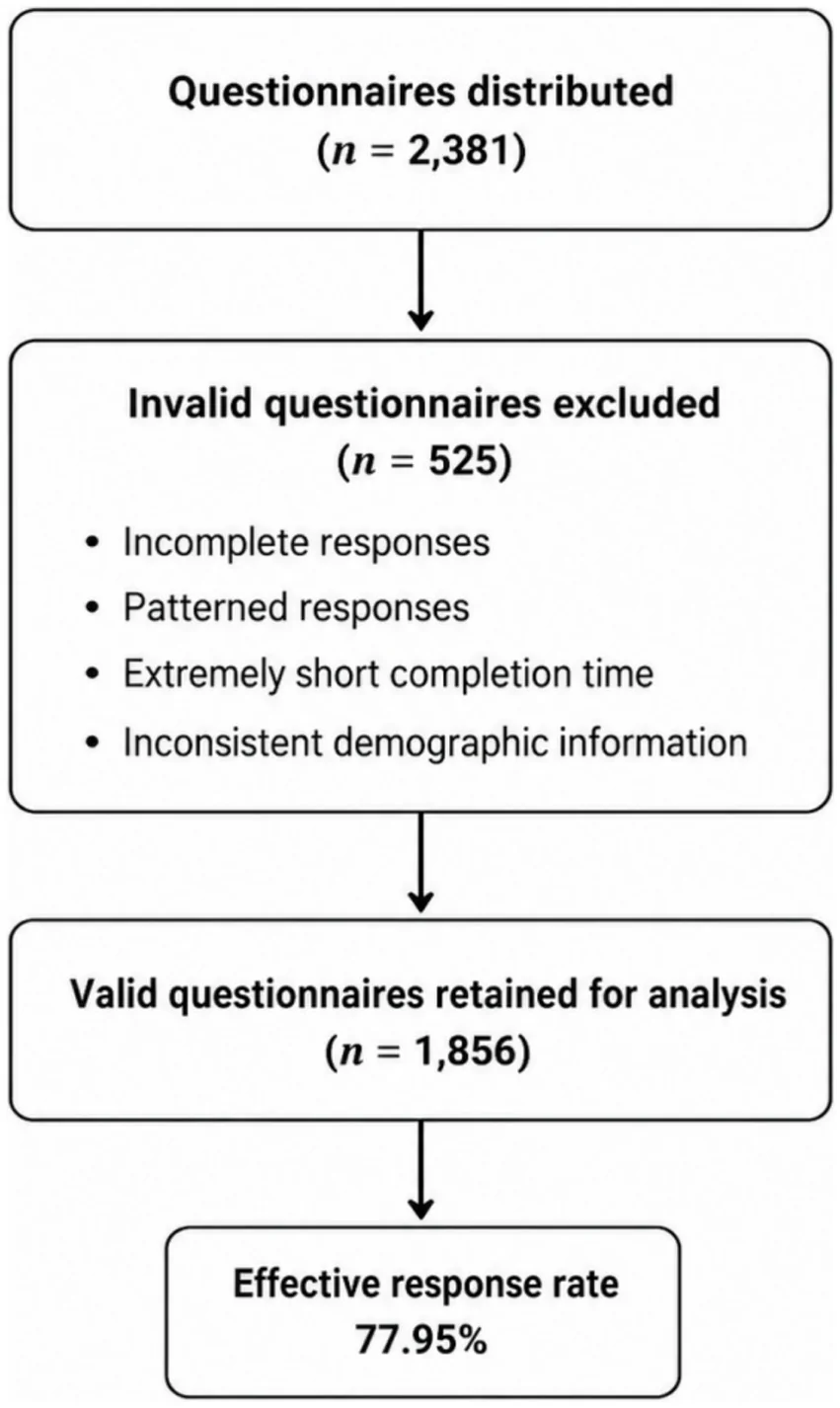
Flow diagram of questionnaire screening and final analytic sample.

The adherence rates for the 24-h movement behavior guidelines are presented in Figure 2. For the individual components, sleep duration showed the highest adherence rate at 58.8%, followed by physical activity at 43.3%. Recreational screen behavior had the lowest adherence rate, with only 30.7% of participants meeting the guideline (Figure 2a). Regarding the total number of guidelines met, 21.2% of participants met none of the guidelines, 36.0% met one guideline, 31.6% met two guidelines, and 11.2% met all three guidelines (Figure 2b). These findings indicate that complete adherence to all three 24-h movement behavior guidelines was relatively low among the university students, whereas partial adherence was more common.

**Figure 2:**
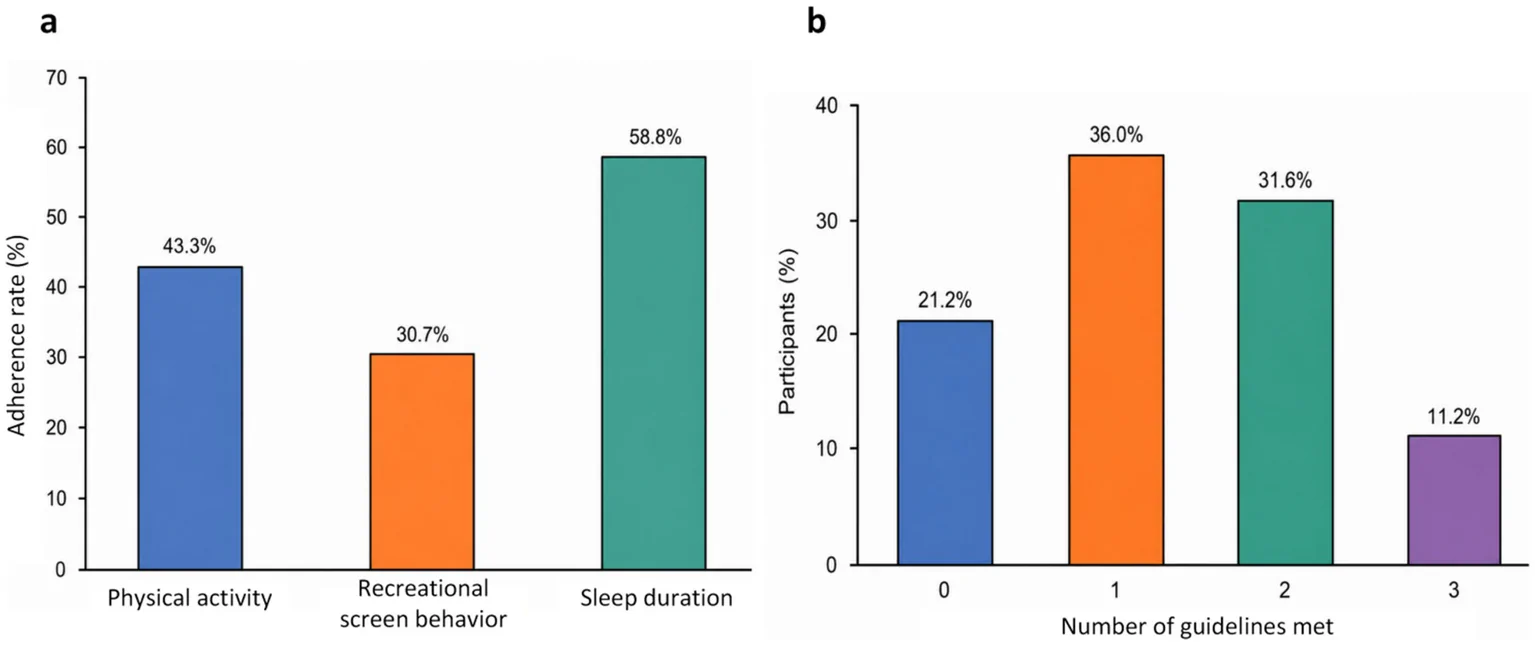
Adherence to 24-h Movement Behavior Guidelines among university students. **(a)** Adherence rates for individual components: physical activity, recreational screen behavior, and sleep duration. **(b)** Distribution of participants meeting 0, 1, 2, or 3 components of the 24-h Movement Behavior Guidelines.

### 4.3 Reliability analysis of the scales

The internal consistency reliability of the main multi-item scales was examined using Cronbach’s alpha coefficients. The results showed that the Cronbach’s alpha coefficient for university adaptation was 0.821, that for psychological resilience was 0.834, and that for positive mental health was 0.747. All Cronbach’s alpha coefficients exceeded the commonly recommended threshold of 0.70, indicating acceptable to good internal consistency and supporting their use in the subsequent correlation and serial indirect-effects analyses.

### 4.4 Correlation analysis

To provide a clearer overview of the hypothesized relationships among the main variables, the research hypothesis model is presented in Figure 3. In this model, adherence to 24-h movement behavior guidelines was specified as the independent variable, university adaptation and psychological resilience were specified as two sequential intervening variables, and positive mental health was specified as the dependent variable. Specifically, H1 proposed an association between guideline adherence and positive mental health. H2 and H3 proposed statistical indirect associations through university adaptation and psychological resilience, respectively. H4 further proposed a serial indirect association through university adaptation and psychological resilience. The ordering shown in the model reflects the *a priori* theoretical hypothesis and does not establish temporal precedence.

**Figure 3:**
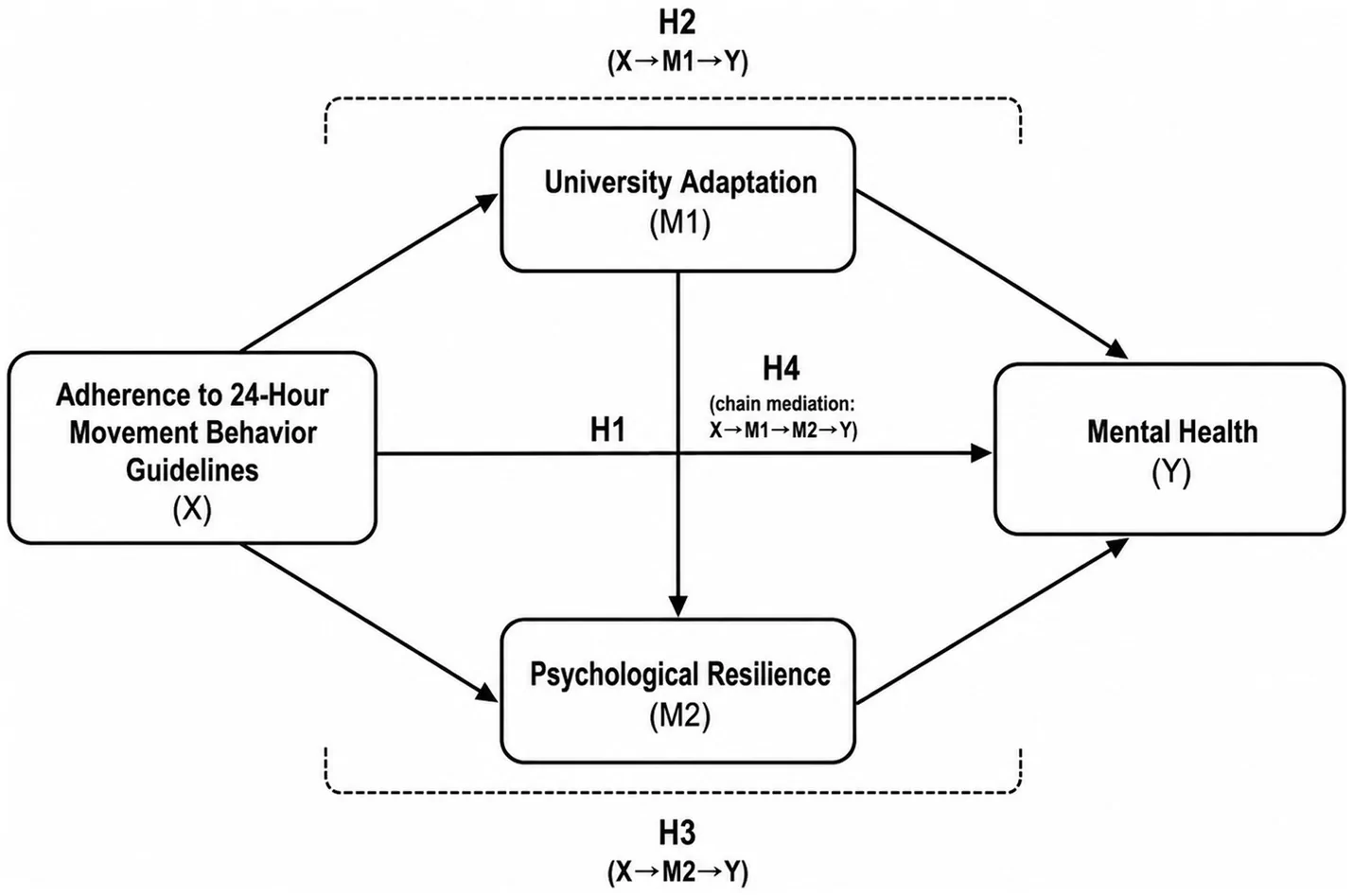
Research hypothesis model. *X* = adherence to 24-h movement behavior guidelines; M1 = university adaptation; M2 = psychological resilience; Y = MHC-SF positive mental health. H1 represents the hypothesized association from *X* to *Y*. H2 represents the hypothesized statistical indirect association through university adaptation. H3 represents the hypothesized statistical indirect association through psychological resilience. H4 represents the hypothesized serial indirect association from *X* through M1 and M2 to *Y*. The ordering is theoretically specified and should not be interpreted as an empirically established temporal sequence.

Pearson correlation analysis was then conducted to examine the associations among the main variables. The descriptive statistics, reliability coefficients, and correlation coefficients are shown in Table 4. Guideline adherence was significantly and positively correlated with university adaptation, psychological resilience, and positive mental health. University adaptation was also significantly and positively correlated with psychological resilience and positive mental health, and psychological resilience was positively correlated with positive mental health. These findings were consistent with the hypothesized pattern of associations and provided a basis for further examination of statistical direct and indirect associations in the subsequent PROCESS analysis.

### 4.5 Serial indirect-effects analysis

To further estimate the statistical indirect associations through university adaptation and psychological resilience, PROCESS Model 6 was applied. Guideline adherence was entered as the independent variable, university adaptation as the first intervening variable (M1), psychological resilience as the second intervening variable (M2), and MHC-SF positive mental health as the dependent variable. Gender, age, grade, university, major, residence, only-child status, family economic status, and BMI were included as covariates. The specified ordering reflects the hypothesized theoretical model rather than an empirically demonstrated temporal sequence.

#### 4.5.1 Regression path coefficients

The regression results of the serial indirect-effects model are presented in Table 5. Adherence to 24-h movement behavior guidelines was significantly and positively associated with university adaptation (*B* = 0.212, *β* = 0.318, SE = 0.015, *p* < 0.001), indicating that students who met more guideline thresholds tended to report better adaptation to university life. The model explained 11.0% of the variance in university adaptation (*R*^2^ = 0.110).

**Table 5:** Regression results of the serial indirect-effects model.

Outcome variable	Predictor	*B*	*β*	SE	*t*	*p*
University adaptation	24-h movement behavior guideline adherence	0.212	0.318	0.015	14.133	<0.001
Psychological resilience	24-h movement behavior guideline adherence	0.096	0.154	0.018	5.333	<0.001
Psychological resilience	University adaptation	0.463	0.495	0.025	18.520	<0.001
Positive mental health	24-h movement behavior guideline adherence	0.082	0.114	0.019	4.316	<0.001
Positive mental health	University adaptation	0.269	0.249	0.024	11.208	<0.001
Positive mental health	Psychological resilience	0.479	0.415	0.025	19.160	<0.001

B = unstandardized regression coefficient; β = standardized regression coefficient; SE = standard error. Standardized coefficients were calculated from B and the sample standard deviations in Table 4. Gender, age, grade, university, major, residence, only-child status, family economic status, and BMI were controlled in the model.

In the equation predicting psychological resilience, guideline adherence was significantly and positively associated with psychological resilience (*B* = 0.096, *β* = 0.154, SE = 0.018, *p* < 0.001). University adaptation was also positively associated with psychological resilience (*B* = 0.463, *β* = 0.495, SE = 0.025, *p* < 0.001), with the latter association showing the larger standardized magnitude. The model explained 32.4% of the variance in psychological resilience (*R*^2^ = 0.324).

When positive mental health was entered as the dependent variable, guideline adherence remained significantly and positively associated with the outcome (*B* = 0.082, *β* = 0.114, SE = 0.019, *p* < 0.001). University adaptation (*B* = 0.269, *β* = 0.249, SE = 0.024, *p* < 0.001) and psychological resilience (*B* = 0.479, *β* = 0.415, SE = 0.025, *p* < 0.001) were also positively associated with positive mental health. These results indicate direct and indirect statistical associations within the specified model rather than temporal mediation. The model explained 40.7% of the variance in positive mental health (*R*^2^ = 0.407).

#### 4.5.2 Bootstrap analysis of indirect effects

The bootstrap results for the direct and indirect effects are shown in Table 6. The total statistical association between guideline adherence and positive mental health was significant (Effect = 0.232, 95% Boot CI [0.184, 0.281]). After university adaptation and psychological resilience were included in the model, the direct statistical association remained significant (Effect = 0.082, 95% Boot CI [0.045, 0.119]), indicating that a direct association remained after accounting for the two intervening variables.

**Table 6:** Bootstrap analysis of direct and indirect effects.

Effect path	Effect	Boot SE	Boot LLCI	Boot ULCI	Conclusion
Total effect: X → Y	0.232	0.025	0.184	0.281	Significant
Direct effect: X → Y	0.082	0.019	0.045	0.119	Significant
Total indirect effect	0.150	0.018	0.116	0.187	Significant
X → M1 → Y	0.057	0.011	0.037	0.080	Significant
X → M2 → Y	0.046	0.010	0.028	0.067	Significant
X → M1 → M2 → Y	0.047	0.009	0.030	0.066	Significant

X = adherence to 24-h movement behavior guidelines; M1 = university adaptation; M2 = psychological resilience; Y = MHC-SF positive mental health. Boot SE = Bootstrap standard error; Boot LLCI = lower limit of the 95% Bootstrap confidence interval; Boot ULCI = upper limit of the 95% Bootstrap confidence interval. An indirect effect was considered significant when the 95% Bootstrap confidence interval did not include zero.

The total indirect effect was also significant (Effect = 0.150, 95% Boot CI [0.116, 0.187]). Regarding the specific indirect paths, a significant statistical indirect association was observed through university adaptation (Effect = 0.057, 95% Boot CI [0.037, 0.080]). A significant statistical indirect association was also observed through psychological resilience (Effect = 0.046, 95% Boot CI [0.028, 0.067]).

In addition, the serial indirect association through university adaptation and psychological resilience was significant (Effect = 0.047, 95% Boot CI [0.030, 0.066]). This statistical pattern was consistent with the hypothesized ordering: meeting more guideline thresholds was associated with better university adaptation, which was associated with higher psychological resilience and better positive mental health. Because all variables were assessed concurrently, however, this result does not establish that these associations occurred in the proposed temporal sequence.

### 4.6 Robustness analysis using categorical guideline adherence

To examine whether the results were robust to the coding of the independent variable, adherence to 24-h movement behavior guidelines was further treated as a categorical variable. Participants were classified into four groups according to the number of guidelines met: meeting no guideline, meeting one guideline, meeting two guidelines, and meeting all three guidelines. The group meeting no guideline was used as the reference group.

The results of the robustness analysis are presented in Table 7. Compared with students who met no guideline, students who met one, two, or all three guidelines showed progressively higher positive mental health scores. The total statistical associations were 0.165, 0.392, and 0.621 for meeting one, two, and all three guidelines, respectively, compared with meeting no guideline. The direct associations also showed a graded pattern across the three comparisons, with coefficients of 0.074, 0.150, and 0.232, respectively.

**Table 7:** Robustness analysis using categorical guideline adherence.

Comparison	Total effect B	Direct effect B	Total indirect effect (95% Boot CI)	Serial indirect effect via M1 → M2 (95% Boot CI)
Met 1 guideline vs. met no guideline	0.165***	0.074*	0.091 [0.049, 0.143]	0.027 [0.011, 0.049]
Met 2 guidelines vs. met no guideline	0.392***	0.150***	0.242 [0.184, 0.308]	0.079 [0.047, 0.114]
Met all 3 guidelines vs. met no guideline	0.621***	0.232***	0.389 [0.293, 0.495]	0.131 [0.082, 0.185]

The reference group was participants who met no 24-h movement behavior guideline. M1 = university adaptation; M2 = psychological resilience. Boot CI = bootstrap confidence interval. The total indirect effect sums the specific indirect associations through M1, through M2, and through M1 → M2. The serial indirect effect refers specifically to X → M1 → M2 → Y. *p < 0.05, ***p < 0.001 for the total and direct effects.

The total indirect effects were statistically significant in all three comparisons, as the 95% bootstrap confidence intervals did not include zero. Specifically, the total indirect effects were 0.091 for meeting one guideline, 0.242 for meeting two guidelines, and 0.389 for meeting all three guidelines, compared with meeting no guideline. These results show that the statistical indirect associations through university adaptation and psychological resilience remained significant when guideline adherence was treated as a categorical variable.

The serial indirect associations through university adaptation and psychological resilience were also statistically significant across all three comparisons. The serial indirect effects were 0.027 for meeting one guideline, 0.079 for meeting two guidelines, and 0.131 for meeting all three guidelines, with all corresponding 95% bootstrap confidence intervals excluding zero. These findings indicate that the statistical pattern of serial indirect associations was robust across different levels of guideline adherence, without establishing temporal or causal ordering.

## 5 Discussion

### 5.1 Main findings

This study examined the association between 24-h movement behavior guideline adherence and positive mental health among university students, together with hypothesized statistical indirect associations through university adaptation and psychological resilience. The principal contribution is not simply to replicate the already documented association between 24-h movement behaviors and mental-health-related outcomes, but to extend this literature by evaluating university adaptation and psychological resilience as potential psychosocial correlates within a theoretically specified serial model. Because the MHC-SF measures emotional, social, and psychological well-being, the outcome in this study represents positive mental health rather than the absence of depression, anxiety, or other psychiatric symptoms.

The correlation analysis showed that greater guideline adherence was positively associated with university adaptation, psychological resilience, and positive mental health. Further PROCESS analysis identified significant direct and statistical indirect associations with positive mental health through university adaptation and psychological resilience. The categorical robustness analysis produced generally consistent findings, suggesting that the main statistical pattern was not dependent on treating the 0–3 adherence count as a continuous variable. These findings are consistent with, but do not establish, the hypothesized psychosocial pathway.

Although the observed associations were statistically significant, their magnitude should also be considered. The correlations between guideline adherence and university adaptation, psychological resilience, and positive mental health were modest (*r* = 0.318, 0.286, and 0.301, respectively), suggesting small-to-moderate associations rather than strong relationships. The specific indirect effects through university adaptation (Effect = 0.057), psychological resilience (Effect = 0.046), and their serial pathway (Effect = 0.047) were also relatively modest in magnitude. Thus, although these associations were statistically reliable, partly owing to the relatively large sample size, they should not be interpreted as indicating large practical or clinical effects. Rather, guideline adherence should be viewed as one of multiple factors statistically associated with university students’ positive mental health.

### 5.2 Adherence to 24-h movement behavior guidelines and positive mental health

The finding that greater guideline adherence was positively associated with MHC-SF positive mental health is consistent with the broader literature linking daily movement behaviors to psychological functioning. Prior university-student studies have primarily examined depression, anxiety, stress, or related symptom outcomes, whereas the present study focuses on positive emotional, social, and psychological well-being. Recent compositional evidence in Chinese college students further suggests that moderate-to-vigorous physical activity, sedentary time, and sleep can show different associations across depression, anxiety, and stress outcomes (Li et al., 2026). Thus, the present positive-mental-health finding should be interpreted as complementary to, rather than equivalent to, evidence on psychiatric or distress symptoms.

Importantly, this study examined physical activity, recreational screen behavior, and sleep duration within an integrated 24-h movement framework. This perspective recognizes that daily behaviors are interrelated, but it should not be taken to imply that all components contribute equally or that a single universal combination is optimal. The emerging precision 24-h movement recommendation perspective emphasizes that optimal behavioral configurations may vary across populations and outcomes (Tremblay et al., 2024). Accordingly, the present 0–3 score is best understood as an index of the breadth of guideline attainment; it does not identify the specific configuration, quality, or relative contribution of the behaviors within the 24-h day.

### 5.3 Statistical indirect associations through university adaptation and psychological resilience

The analysis identified significant statistical indirect associations through both university adaptation and psychological resilience. Students who met more guideline thresholds tended to report better university adaptation, and better adaptation was associated with better positive mental health. One theoretical possibility is that healthier daily routines co-occur with differences in energy, attention, emotional stability, or participation that are relevant to university adjustment. However, these intermediate processes were not measured, and they should therefore be regarded as hypotheses for future research rather than demonstrated mechanisms.

More importantly, a significant serial indirect association was observed through university adaptation and psychological resilience. The statistical model was specified in the theoretically hypothesized order of guideline adherence → university adaptation → psychological resilience → positive mental health. This ordering is plausible because successful adjustment to university demands may provide experiences of competence, belonging, social support, and coping that are associated with resilience. Nevertheless, the reverse explanation is equally plausible: more resilient students may adapt more effectively to university life, and reciprocal relationships may operate over time. The cross-sectional data therefore cannot establish that the variables occurred in the proposed temporal sequence. The serial indirect effect should be interpreted only as a statistical association consistent with the hypothesized model, not as evidence of a mediation mechanism.

### 5.4 Practical implications, limitations, and future directions

The findings may inform the design of future university health-promotion research. Integrated programs could consider physical activity, recreational screen-time management, sleep education, university adjustment support, and resilience-related skills within a broader framework. However, the present cross-sectional associations do not establish that changing any of these factors will improve positive mental health. These elements should therefore be viewed as potentially relevant components for prospective intervention development and testing rather than as established causal intervention targets.

Several limitations should be noted. First, the cross-sectional design precludes conclusions about temporal precedence, causal mediation, or directionality; longitudinal and experimental designs are needed to test whether changes in movement behaviors precede changes in university adaptation, resilience, and positive mental health. Second, all three movement components were assessed by self-report and over non-harmonized reference periods: physical activity referred to the previous 7 days, sleep duration to the previous month, and recreational screen behavior to average daily exposure. This mismatch means that the 0–3 score is not a synchronized composition of a single 24-h period. Recent methodological work emphasizes the importance of validated, harmonized assessment across physical activity, sedentary behavior, and sleep, including careful use of wearable devices (Giurgiu et al., 2025). Future studies should therefore consider accelerometry, validated sleep monitoring, and objectively recorded screen exposure collected over a common monitoring window.

Third, recreational screen behavior captures only one aspect of sedentary behavior. The present results should therefore not be interpreted as evidence about total sedentary time or the complete sedentary-behavior component of the 24-h movement guidelines. Fourth, external validity is limited by the convenience sample and its distribution: 1,402 participants were recruited from Hunan First Normal University and 454 from Hunan Normal University, and the age distribution was narrow (19.13 ± 0.56 years). These features may limit generalizability to students from other universities, regions, age groups, or stages of university study.

Fifth, the additive 0–3 adherence score gives equal weight to the three operationalized components and captures only the breadth of guideline attainment. Students with the same score may meet different combinations of physical activity, recreational screen behavior, and sleep, and those configurations may not have equivalent associations with positive mental health. Precision-recommendation and compositional approaches highlight this limitation (Tremblay et al., 2024; Li et al., 2026). Future studies should examine specific guideline combinations and use compositional methods where the measurement design supports a complete 24-h time-use analysis. Finally, although university adaptation was specified before psychological resilience on theoretical grounds, reverse and reciprocal ordering remains plausible and should be tested with longitudinal data.

## 6 Conclusion

This study found modest positive associations between greater breadth of 24-h movement behavior guideline adherence and MHC-SF positive mental health among university students. University adaptation and psychological resilience also showed statistical indirect associations consistent with the hypothesized serial pathway. These findings extend prior 24-h movement research by identifying psychosocial correlates that may help guide future longitudinal testing, but they do not demonstrate temporal mediation or causality. The 0–3 adherence score represents breadth of guideline attainment rather than an optimal behavioral composition, and future research should combine harmonized objective monitoring with analyses of specific movement-behavior configurations and competing temporal pathways.

## Data Availability

The raw data supporting the conclusions of this article will be made available by the authors, without undue reservation.
